# Screening and optimization of indole-3-acetic acid production by *Rhizobium* sp. strain using response surface methodology

**DOI:** 10.1186/s43141-020-00035-9

**Published:** 2020-06-18

**Authors:** Sara Lebrazi, Mouhcine Fadil, Marwa Chraibi, Kawtar Fikri-Benbrahim

**Affiliations:** 1 Laboratory of Microbial Biotechnology, Sciences and Technology Faculty, Sidi Mohamed Ben Abdellah University, P.O. Box 2202, Fez, Morocco; 2 Physico-chemical laboratory of inorganic and organic materials, Materials Science Center (MSC), Ecole Normale Supérieure, Mohammed V University in Rabat, Rabat, Morocco

**Keywords:** *Rhizobium*, Plant growth, Indole-3-acetic acid, Optimization, Experimental design, Response surface methodology

## Abstract

**Background:**

The production of indole-3-acetic acid (IAA) is an essential tool for rhizobacteria to stimulate and facilitate plant growth. For this, eighty rhizobial bacteria isolated from root nodules of *Acacia cyanophylla* grown in different regions of Morocco were firstly screened for their ability to produce IAA. Then, IAA production by a combination of isolates and the inoculation effect on the germination of *Acacia cyanophylla* seeds was studied using the best performing isolates in terms of IAA production. The best IAA producer bacterial isolate (I69) was selected to optimize IAA production using response surface methodology based on the central composite design.

**Results:**

Results showed that the majority of tested isolates were able to produce IAA with a relatively higher concentration of 135 μg/ml for the isolate I69, followed by isolates I22 and I75 with respective concentrations of 116 μg/ml and 105 μg/ml IAA. The IAA production and the seed germination rate were relatively increased by the synergistic effect of I69 and I22. Later, response surface methodology was used to determine optimal operating conditions leading to IAA production optimization. Thus, an incubation temperature of 36 °C, a pH of 6.5, an incubation time of 1 day, and respective tryptophan and NaCl concentrations of 1 g/l and 0.1 g/l were optimal parameters leading to 166 μg/ml IAA which was the maximal produced concentration.

**Conclusion:**

The present study highlighted that IAA-producing rhizobacteria could be harnessed to improve plant growth. Furthermore, their production can be easily controlled using response surface methodology, which represents a very useful tool for optimization.

## Background

Indole-3-Acetic Acid (IAA) is one of the most important and physiologically active phytohormones [1, 2]. It is a secondary metabolite of L-tryptophan that acts as a regulator of many biological processes for plant development while acting on organogenesis, trophic responses, and cellular responses such as cell expansion, division, differentiation, and regulation of genes [3, 4]. The majority of rhizobacteria can produce IAA that is the most abundant type of auxins [5]. Under natural conditions, plant roots excrete organic compounds, including L-Trp that can be used by rhizobacteria for IAA biosynthesis which can help non-native plant species to resist under biotic and abiotic stress conditions [6–8]. However, little information is available on the relationship between stress and auxins in plants, and the evolutionary role played by auxin in adapting plants to various environmental stresses [9, 10].

Indeed, several authors have reported the role of this phytohormone in plants’ adaptation to salinity stress [11, 12] and heavy metal stresses [13]. IAA has been well documented as an essential phytohormone known primarily for its ability to stimulate plant growth and development [6, 14]. Indeed, IAA synthesized by rhizobacteria affects mostly the root system by increasing its size, weight, lateral roots number, and the area of contact with the soil. This mechanism contributes to increase nutrient research and acquisition in soil, which improves plant development and yield [15, 16]. Moreover, IAA can act as a reciprocal signaling molecule by affecting gene expression in many bacteria and also plays a critical role in the plant-bacteria interaction [17, 18]. Also, it has been shown that nodulated roots contain more IAA than non-nodulated roots [19, 20], and auxins could be essential for maintaining a root nodule functional [21].

IAA production by rhizobacteria can differ considerably between different species or strains of the same species. Moreover, several environmental factors can influence the biosynthesis of this phytohormone [22], in particular, a high pH and the presence of large quantities of tryptophan, which lead to an increase in its production [21]. In fact, Chandra et al. [23] found that their tested isolates for optimizing the IAA production showed better amount of IAA produced at pH 9. Similarly, Shoukry et al. [24] reported that pH 7 was the optimum growth pH for IAA production by *Rhizobium* strains in medium supplemented with 5 g/l L-tryptophan.

This study aimed to perform a screening of rhizobacteria that produce IAA and to optimize factors leading to the highest IAA concentration using experimental designs methodology. These methods allow experimentation with a minimum number of experiments and give the possibility of screening for different factors [25] from the most influential to the least influential. Also, it makes possible the optimization of the operating conditions giving the best possible result.

Several studies have addressed the topic of IAA production optimization. Some of them have used the classical optimization approach, which consists in studying the effect of each factor separately while fixing the others [24, 26, 27]. Others have used designs of experiments type Taguchi [28] and Plackett and Burman [29]. The use of experimental designs in optimization has shown many benefits compared to the classical approach because it gives the possibility of modeling responses and studying the interaction between factors. In this way, we have chosen to use a response surface design because it is better recommended than the Plackett and Burman designs and Taguchi tables since it concerns an optimization rather than a factor screening.

## Methods

### Isolation of rhizobia from nodules

Nodules were harvested, according to the method recommended by Vincent [30] and Beck et al. [31], from the roots of *Acacia cyanophylla* from three Moroccan regions (Eastern, Central, and North West). These nodules were immersed in 95% (v/v) ethanol for 30 s, then transferred to a mercury chloride (HgCl_2_) solution 0.1% for 4 min. A series of three rinses of 10, 15, and 20 min, respectively, were performed under aseptic conditions using sterile distilled water [30, 32]. Surface-sterilized nodules were crushed using a few drops of NaCl (9 ‰) [31]. The operation was performed under conditions of total asepsis. One hundred microliters of the suspension obtained was spread on Petri dishes containing Yeast-Mannitol-Agar (YMA) medium [30].

### Screening of isolates for IAA production

Different *Rhizobium* isolates from *Acacia cyanophylla*’s root nodules isolated from three Moroccan regions (Eastern, Central, and North West) were tested for their ability to produce IAA. For this, the amount of IAA production in each isolate was determined by the colorimetric technique by using Salkowski reagent containing: 50 ml, 35% perchloric acid (HClO_4_); 1 ml of 0.5 M iron trichloride (FeCl_3_) according to the protocol proposed by Bric et al. [33]. The bacterial isolates were cultured in Erlenmeyer of 250 ml containing 50 ml of YMB supplemented with 2 g/l L-tryptophan, at 28 ± 2 °C for 7 days at a shaking speed of 150 rpm in an orbital shaking incubator. Afterward, bacterial cultures were centrifuged at 10.000 rpm for 10 min at 4 °C, and the supernatant liquid was mixed with Salkowski reagent (1:2). The mix was incubated for 30 min in the dark at 28 ± 2 °C, and then absorbance was measured at 530 nm. The concentration of IAA produced was estimated using a standard IAA curve. All IAA determination experiments were made in triplicate.

### Study of IAA production by a combination of isolates

The IAA production by a combination of the three best producing strains of this acid (*Rhizobium* sp. KX884900.2 (I22), *Rhizobium* sp. KJ748400.1 (I69), and *Agrobacterium rhizogenes*CP019702.1 (I75) was tested. For this, from the primary cultures of the three bacterial strains (10^8^ CFU/ml), four mixed cultures (I22 + I69, I22 + I75, I69 + I75, and I22 + I69 + I75) were prepared and then tested in 50 ml of YMB medium supplemented with L-tryptophan (1 g/l). After incubation at 28 ± 2 °C with 150 rpm for 7 days, cultures were centrifuged, and the IAA concentration produced was measured and estimated for each culture at 530 nm using the Salkowski reagent. Three repetitions were made for each test.

### Effect of inoculation on *A. cyanophylla* seed germination

#### Seed preparation

Tested seeds were obtained from the High Commission for Water and Forests and the Fight against Desertification of Fes-Boulemane region (http://www.eauxetforets.gov.ma). Then, they were surface-sterilized with 70% ethanol (v/v) and immersed in 0.1% (w/v) fresh-made HgCl_2_ for 5–10 min, followed by three washes with sterile distilled water and soaking in 95% H_2_SO_4_ to scarify them [34, 35]. After rinsing with sterile distilled water, seeds were imbibed under sterile conditions for 6 h at room temperature.

### Preparation of the bacterial inoculum and seed bacterization

The three *Rhizobium* isolates, showing the highest IAA productions, were selected to verify their effect on the germination of *A. cyanophylla*’s seeds. These isolates were cultured separately in liquid stirred YMB (150 rpm) at 28 °C for 48 h. Then, the bacterial suspensions were centrifuged at 10,000 rpm for 10 min. The pellet was suspended in 10 ml of MgSO_4_, 7H_2_O (0.1 M), and surface-sterilized seeds were soaked in the bacterial suspension for 30 min. The control seeds were immersed in a magnesium sulfate solution for the same duration [36]. Seeds were subsequently dried in a stream of sterile air for 1 h [37]. Germination was performed in Petri dishes containing 1% agar and incubated in the dark at 28 °C until radicle emergence for 3 to 10 days. The parameters studied in this test are root length and final germination rate, which is expressed as the ratio of the germinated seed number on total seed number.

### Experimental design

Experimental designs present useful tools for parameters’ screening and optimization. Based on the laws of statistical regression, they allow the quantification of various factor effects on a studied response and the optimization of operating conditions in well-defined experimental areas.

The central composite design, which is a part of response surface methodology, is used for response optimization. In this context, a sequence of experiments is carried out to evaluate the effect of factors on response and to obtain the optimal response.

### The fitted model

To express the response as a function of the independent variables, we have used a quadratic model such as Eq. (1):

1 $$ \mathrm{Y}={\mathrm{b}}_0+{\mathrm{b}}_1{\mathrm{X}}_1+{\mathrm{b}}_2{\mathrm{X}}_2+{\mathrm{b}}_3{\mathrm{X}}_3+{\mathrm{b}}_4{\mathrm{X}}_4+{\mathrm{b}}_5{\mathrm{X}}_5+{\mathrm{b}}_{11}{\mathrm{X}}_1{\mathrm{X}}_1+{\mathrm{b}}_{22}{\mathrm{X}}_2{\mathrm{X}}_2+{\mathrm{b}}_{33}{\mathrm{X}}_3{\mathrm{X}}_3+{\mathrm{b}}_{44}{\mathrm{X}}_4{\mathrm{X}}_4+{\mathrm{b}}_{55}{\mathrm{X}}_5{\mathrm{X}}_5+{\mathrm{b}}_{12}{\mathrm{X}}_1{\mathrm{X}}_2+{\mathrm{b}}_{13}{\mathrm{X}}_1{\mathrm{X}}_3+{\mathrm{b}}_{23}{\mathrm{X}}_2{\mathrm{X}}_3+{\mathrm{b}}_{14}{\mathrm{X}}_1{\mathrm{X}}_4+{\mathrm{b}}_{24}{\mathrm{X}}_2{\mathrm{X}}_4+{\mathrm{b}}_{34}{\mathrm{X}}_3{\mathrm{X}}_4+{\mathrm{b}}_{15}{\mathrm{X}}_1{\mathrm{X}}_5+{\mathrm{b}}_{25}{\mathrm{X}}_2{\mathrm{X}}_5+{\mathrm{b}}_{35}{\mathrm{X}}_3{\mathrm{X}}_5+{\mathrm{b}}_{45}{\mathrm{X}}_4{\mathrm{X}}_5+\upvarepsilon $$

with Y = the response (IAA) expressed in (μg/ml); b_0_ is the constant term; b_1_, b_2_b_3_ b_4_ b_5_ are coefficients of the main terms; b_11_, b_22_, b_33_, b_44,_ and b_55_ are coefficients of quadratic terms; and b_12_, b_13_, b_23_; b_14_, b_24_, b_34_, b_15_, b_25_, b_35_ and b_45_ are coefficients of binary terms

### Statistical analysis

The second-order model coefficients were calculated based on the experimental data. The statistical analysis was carried out using ANOVA test. In this way, the ratio between the mean square regression (MSR) and the mean square residual (MSr), *F*_ratio(R/r)_, was used at a 95% significance level to check the model statistical significance [38].

### Optimization of IAA production by experimental design

Among all tested isolates, the best IAA producer was selected to study the effect of different variables on this phytohormone’s production. Later, its optimization based on response surface methodology-central composite design was investigated. The chosen variables were L-tryptophan and NaCl concentrations, pH, incubation time, and incubation temperature. All factors were studied at three different levels (Table 1).

**Table 1:** Factors and their levels used in the optimization of IAA production by *Rhizobium sp.* using the central composite design

Symbol	Variables	Level 1	Level 2	Level 3
**X1**	[L-tryptophan] (g/l)	0.1	0.5	0.9
**X2**	[NaCl] (g/l)	0	2.5	5
**X3**	Temperature (°C)	30	36	42
**X4**	pH	4	6.5	9
**X5**	Incubation time (days)	1	8	15

## Results

### Screening of isolates for IAA production

The colorimetric assay showed that 96% of the eighty tested isolates were able to produce IAA on YMB medium at varying concentrations depending on their efficiency and their enzymatic potency. Indeed, a relatively higher concentration was found in the culture filtrate of strain I69 (135 μg/ml).

### Study of IAA production by a combination of isolates

The combined effect of the three best IAA producers (I22, I69, and I75) was studied.

**Fig. 1:**
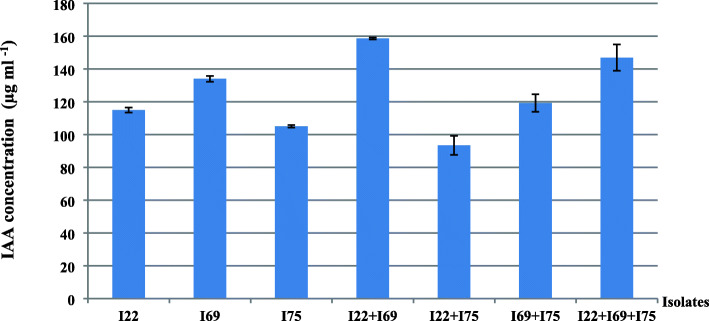
Production of IAA by the combination of three rhizobial isolates (I22, I69, and I75)

The obtained results revealed that the combination of two isolates I22 and I69 induces the highest hormonal production with 158.68 μg/ml of IAA (Fig. 1). However, the inoculation by I75, in combination with the other two isolates, showed a negative effect on IAA production.

### Inoculation effect on *Acacia cyanophylla* seed germination

**Fig. 2:**
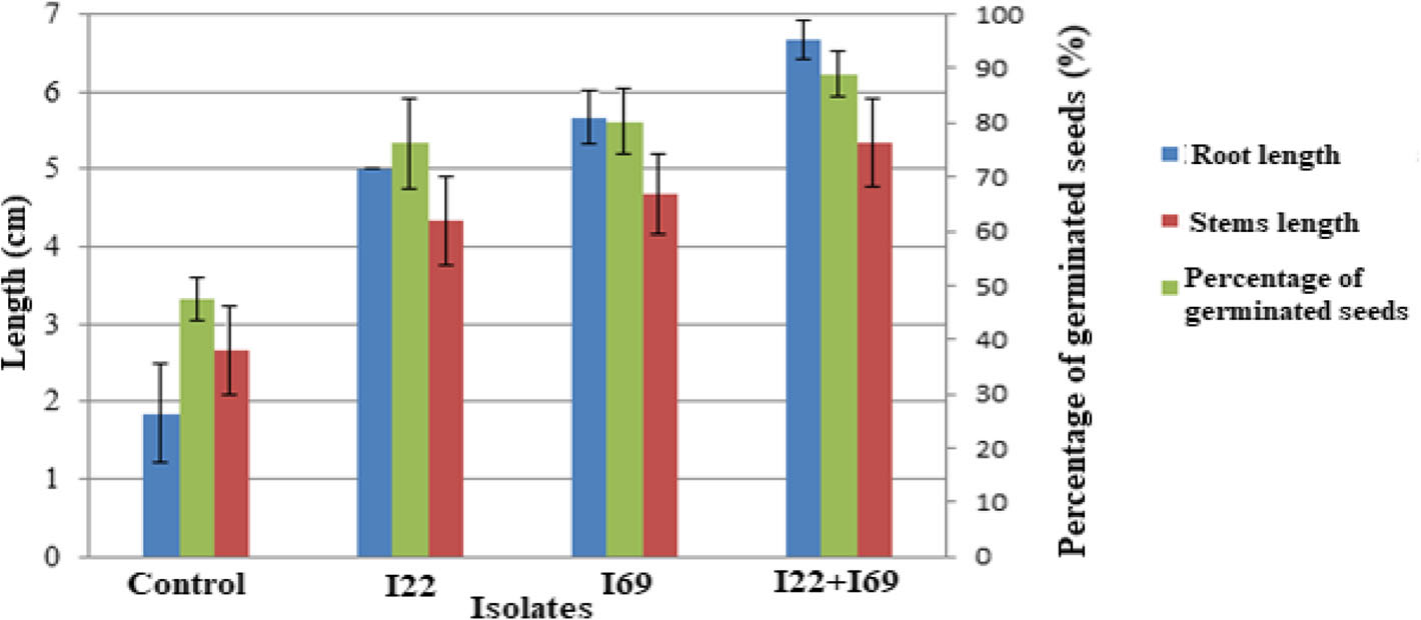
Inoculation effect by the isolates I22 and I69 on seed germination of *Acacia cyanophylla* and seedling growth

The two isolates I22 and I69 having the ability of synergistic IAA biosynthesis were further tested for their impact on seed germination of *A. cyanophylla* and seedling growth. The observations were made under controlled conditions in Petri dishes, where the effect of the tested bacteria on the plant’s growth is a direct consequence of its interaction with the plant. Seed bacterization by both of the studied isolates (Fig. 2) showed their ability to form a beneficial association with the *A. cyanophylla* seedlings. Isolates I22 and I69 were able to increase the germination rate by 1.61 and 1.68 times, respectively, compared to control, while the submission of seeds to a combination of these two isolates showed a germination rate increase of 1.87 times. Thus, stimulation of stem length and seedling roots has been demonstrated by our isolates. Respective increases of stem length of 1.62, 1.75, and 2 times were observed for inoculation treatments with I22, I69, and I22 + I69, while root length increases of 2.72, 3.09, and 3.63 times were respectively obtained for the three inoculation treatments. The results obtained during this study showed a significant effect of isolates on seedling germination and growth (*p* < 0.001).

### Optimization of IAA production by central composite design

The studied isolates were screened for their ability to produce IAA. Results showed that 77 isolates among the 80 tested ones were able to produce this phytohormone. The isolate I69 leads to the highest IAA production (135 μg/ml). The effects of various factors on IAA production by I69 and its optimization were studied using a central composite design containing 30 experiments. The experimental design, including the different combinations between studied factors and observed responses for each experiment, is shown in Table 2.

**Table 2:** Experimental plan for the optimization of IAA production by the strain I69: experiment 27, 28, 29, and 30 represent central points

N°Exp	Incubation temperature (°C)	pH	[NaCl] (g/l)	[Tryptophane] (g/l)	Incubation time (days)	[IAA] (μg/ml)
1	30	4.0	0.10	1	15	6.90
2	42	4.0	0.10	1	1	6.34
3	30	9.0	0.10	1	1	18.87
4	42	9.0	0.10	1	15	4.91
5	30	4.0	1.00	1	1	8.25
6	42	4.0	1.00	1	15	2.76
7	30	9.0	1.00	1	15	11.34
8	42	9.0	1.00	1	1	6.58
9	30	4.0	0.10	9	1	5.00
10	42	4.0	0.10	9	15	5.80
11	30	9.0	0.10	9	15	6.96
12	42	9.0	0.10	9	1	4.03
13	30	4.0	1.00	9	15	4.30
14	42	4.0	1.00	9	1	3.76
15	30	9.0	1.00	9	1	27.84
16	42	9.0	1.00	9	15	32.41
17	30	6.5	0.55	5	8	90.72
18	42	6.5	0.55	5	8	2.55
19	36	4.0	0.55	5	8	2.98
20	36	9.0	0.55	5	8	16.58
21	36	6.5	0.10	5	8	119.73
22	36	6.5	1.00	5	8	123.27
23	36	6.5	0.55	1	8	157.79
24	36	6.5	0.55	9	8	108.14
25	36	6.5	0.55	5	1	121.93
26	36	6.5	0.55	5	15	88.92
27	36	6.5	0.55	5	8	118.19
28	36	6.5	0.55	5	8	118.27
29	36	6.5	0.55	5	8	117.66
30	36	6.5	0.55	5	8	117.55

### Statistical validation of the postulated model

**Fig. 3:**
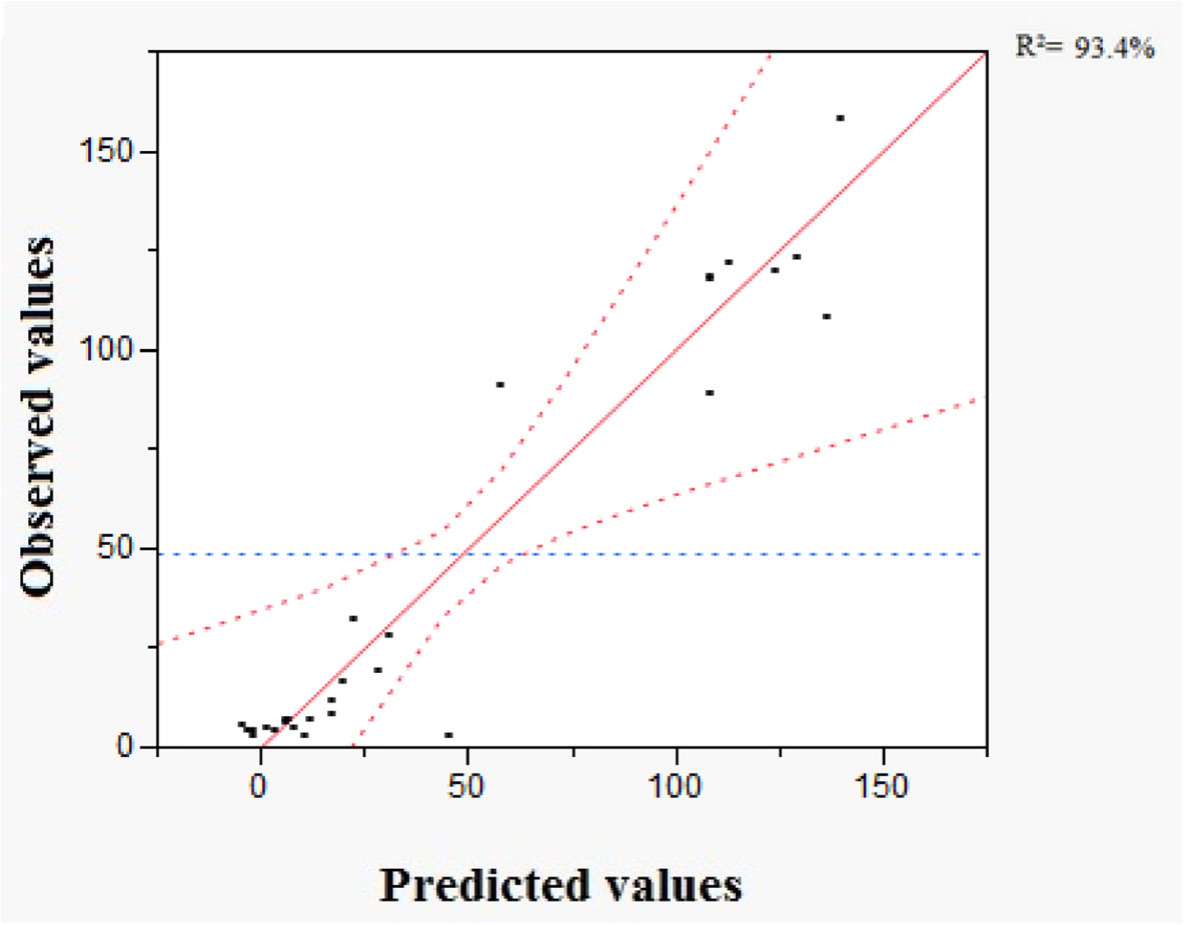
Curve of observed values in term of predicted ones

Variance analysis shows that the main regression effect is significant since the *p* value is lower than 0.05 (Table 3). The coefficient of determination *R*^2^ (93.4%) was sufficient and gave excellent compatibility between the experimental and predicted values of the postulated model. Figure 3 confirms that the curve of observed values versus predicted ones has a straight line appearance.

**Table 3:** Analysis of variance for the fitted model

Source of variance	DF	SS	MS	*F*_ratio_	*p* value
*R*	20	79056.424	3952.82	6.3636	0.0036*
*r*	9	5590.451	621.16		
Total	29	84646.875			
*R*^2^	93%				

*R* regression, *r* residual, *SS* sum of squares, *DF* degrees of freedom, *MS* mean square, *R*^2^ coefficient of determination

*Statistically significant at *p* < 0.05 probability

### Factors effects and fitted model

Table 4 presents the different estimated coefficients of the studied factors as well as the statistical values of Student’s *t* and the observed probability (*p* value). The values of Student’s test are used to determine the significance of each parameter, while *p* values are defined as the lowest level of importance, leading to the rejection of null hypothesis H_0_ (bi = 0, α = 0.05). In general, the greater is the magnitude of *t*, the smaller is the *p* value, and the larger is the coefficient corresponding term. Regarding those results (Table 4), the statistically significant coefficients were as follows:

**Table 4:** Effects of model coefficients that relate the response to factors

Term	Coefficient	Estimation	Erreur standard	Report ***t***	Prob.>|***t***|
Constant	b_0_	107.99474	7.786987	13.87	< 0.0001***
Incubation temperature (30,42)	b_1_	− 6.168889	5.874433	− 1.05	0.321
pH(4,9)	b_2_	4.635	5.874433	0.79	0.4504
[NaCl](0,1,1)	b_3_	2.3316667	5.874433	0.4	0.7007
[Tryptophane](1,9)	b_4_	− 1.416667	5.874433	− 0.24	0.8148
Incubation time (1,15)	b_5_	− 2.127778	5.874433	− 0.36	0.7256
Incubation temperature *pH	b_12_	− 0.705625	6.230777	− 0.11	0.9123
Incubation temperature *[NaCl]	b_13_	0.651875	6.230777	0.1	0.919
pH*[NaCl]	b_23_	3.023125	6.230777	0.49	0.6391
Incubation temperature *[Tryptophane]	b_14_	1.666875	6.230777	0.27	0.7951
pH*[Tryptophane]	b_24_	2.183125	6.230777	0.35	0.7341
[NaCl]*[Tryptophane]	b_34_	3.413125	6.230777	0.55	0.5972
Incubation temperature *Incubation time	b_15_	3.476875	6.230777	0.56	0.5904
pH*Incubation time	b_25_	0.118125	6.230777	0.02	0.9853
[NaCl]*Incubation time	b_35_	0.878125	6.230777	0.14	0.891
[Tryptophane]*Incubation time	b_45_	1.435625	6.230777	0.23	0.8229
Incubation temperature * Incubation temperature	b_11_	− 56.39836	15.90287	− 3.55	0.0063**
pH * pH	b_22_	− 93.25336	15.90287	− 5.86	0.0002***
[NaCl]*[NaCl]	b_33_	18.466643	15.90287	1.16	0.2754
[Tryptophane]*[Tryptophane]	b_44_	29.931643	15.90287	1.88	0.0925
Incubation time*Incubation time	b_55_	2.3916429	15.90287	0.15	0.8838

**Statistically significant at *p* < 0.01 probability

***Statistically significant at *p* < 0.001 probability

- The constant b_0_

- The quadratic terms b_11_, b_22_.

All these factors have a *p* value less than 0.05.

The mathematical model representing the response in terms of significant coefficients is represented by the following equation (Eq. (2)):

2 $$ \hat{Y}=108-56,39{X}_{11}^2-93,25{X}_{22}^2 $$

### Optimization of parameters

#### Isoresponses plot

**Fig. 4:**
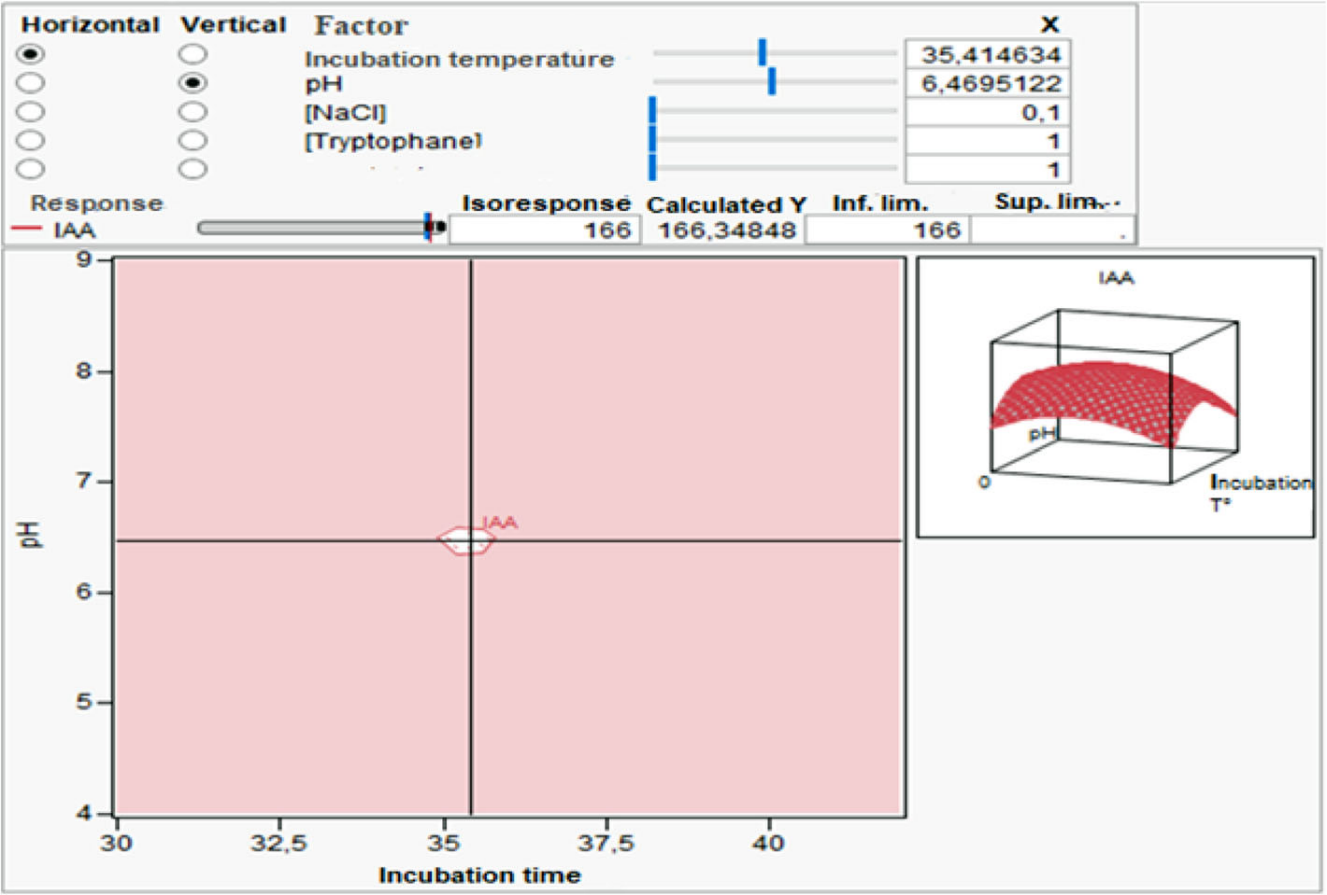
Isoresponses plot for the IAA production optimization by considering both incubation temperature and pH factors and by fixing the concentrations of L-Trp at 1 g/l and NaCl at 0.1 g/l and the incubation time at 1 day

The isoresponse profile plot (Fig. 4) allowed us to consider various solutions relating to the operating parameters.

According to literature, the maximum production of IAA was reached after 9 days of incubation with a L-Trp concentration of 2 g/l [3]. Thus, the objective of our study was to optimize the studied parameters to produce the highest amount of IAA by I69 while minimizing incubation time and concentration of L-Trp added to growth medium.

Considering the isoresponse plot (Fig. 4), the minimum incubation time to reach the desired yield (166 μg/ml) is 1 day. For this period, L-Trp and NaCl concentrations giving this yield are respectively 1 g/l and 0.1 g/l. The next step is to fix these values for the three previous parameters and look for the values of the two other factors.

The white area in isoresponse plot (Fig. 4) shows a compromise zone for both parameters: incubation temperature and pH to obtain the desired concentration of IAA by isolate I69 after fixing the other three parameters at their optimal level. The fixation of these parameters (concentration of L-Trp and NaCl and incubation time) allowed us to know the domains of variation of the other factors: temperature of incubation and pH. Hence, obtaining a concentration of 166 μg IAA/ml requires an incubation period of 1 day, respective concentrations of L-Trp and NaCl of 1 g/l and 0.1 g/l, a pH value between 6.19 and 6.74, and an incubation temperature between 34.46 and 36.18 °C.

The desirability study allowed us to define precisely the optimized values of the five studied factors.

#### Desirability study

**Fig. 5:**
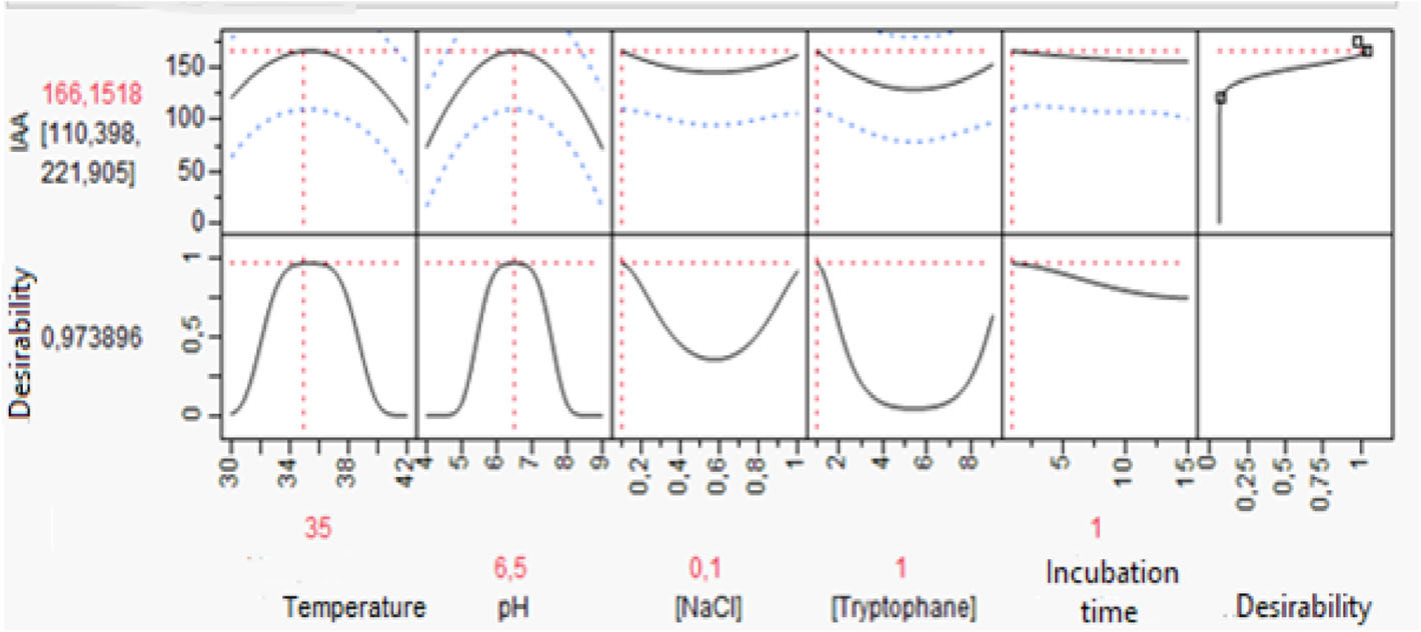
Desirability plot showing the precise operating conditions leading to the optimal yield of IAA production

This optimization tool allowed us to know precisely the optimal values of studied parameters, leading to the expected yield with a defined degree of compromise (desirability). The desirability plot of optimal IAA production conditions is shown in Fig. 5.

This plot indicates that it is possible to achieve an optimal IAA yield with relative desirability equal to 98%. Thus, parameters giving the best IAA production are an incubation temperature of 36 °C, a pH of 6.5, respective L-Trp, NaCl concentrations of 1 g/l and 0.1 g/l, and an incubation time of 1 day. With these conditions, we have a 98% probability to produce IAA at a concentration of 166 μg IAA/ml.

## Discussion

The variation in IAA production by different rhizobial isolates and the influence of cultural conditions on IAA produced amount has been reported by many authors [39–41]. The variation in IAA production is may be due to variation in L-Trp utilization by each isolate. Our results revealed that IAA production by the tested isolates was much higher than those found in previous studies. Indeed, the maximal reported production amounts were 80.96 μg IAA/ml, 99.7 μg AIA/ml, 107 μg IAA/ml, and 142 μg IAA/ml by *Rhizobium* sp. isolated respectively from root nodules of *Vigna trilobata* (L) Verdc., *Cajanus cajan*, *Alysicarpus vaginalis* DC, and *Phaseolus mungo* [42–45].

The IAA production by a combination of *Rhizobium*, *Agrobacterium*, and *Paenibacillus* was studied by Shokri and Emtiazi [28]. These researchers found an increase in IAA concentration produced by *Rhizobium* and *Paenibacillus* combination, showing a synergistic effect. But this concentration decreased when *Agrobacterium* was used in combination with the other two bacteria. Similar results were obtained by El-Shanshoury [46], who found that double inoculation with *Azotobacter chroococcum* and *Azospirillum brasilense* or *Streptomyces mutabilisa* significantly stimulated IAA production.

The reduction in IAA production can be due to the release of enzymes degrading this hormone, such as IAA oxidase and peroxidase, as already reported by several authors [28, 43, 47]. Other studies have reported the ability of rhizobia to produce these enzymes in the medium inducing reduction of hormone concentration [43, 48]. Incompatibility of I75 with I22 and I69 may be due to competition between these bacterial strains and to production of some compounds inhibiting bacterial growth. This antagonistic effect was previously explained by nutritive competition phenomena when bacterial density is high [49, 50].

Our results concerning the effect of inoculation by the tested bacteria on *Acacia cyanophylla* seed germination are consistent with several studies [26, 41, 44] which claimed that the use of PGPR specifically IAA producing bacteria might have a significant effect on increasing seed germination rate and promoting plant growth.

It was shown that longer root systems provide better access to stored water and nutrients such as nitrogen, a soluble nutrient that tends to seep into the deeper soil layers [51]. IAA controls a wide variety of processes in plant growth and root system development. Indeed, low concentrations of IAA can stimulate primary root elongation, while higher levels of this hormone stimulate lateral root formation, decrease primary root length, and increase absorbent hair formation [52]. Seed germination in any plant species is an active metabolic process that begins when seeds are exposed to an appropriate temperature and humidity [53]. IAA has been known for its participation in the early stages of germination in certain plant seedlings [54]. The synthesis of plant growth regulators, in particular IAA, initiates germination of the plants’ seeds, but initiation is triggered by germination stimulators released from host plant roots [53].

The experimental design was a powerful tool that has been used successfully to test the relative importance of environmental factors in IAA production. Shokri and Emtiazi [28] revealed a maximum IAA production by *Rhizobium* sp. at a concentration of 3 g/l of L-Trp after 3 days of incubation at 30 °C. A similar study by Leong [55] mentioned that the optimal amount of IAA produced by *Rhodopseudomonas palutris* was obtained after 2 days of incubation at 35 °C in the presence of 5 g/l L-Trp. Similarly, Ghosh and Basu [56] showed that *Rhizobium* spp. isolated from root nodules of *Dalbergia lanceolaria* produced a maximum amount of IAA at a concentration of 2.5 g/l L-Trp. The use of experimental design allowed us to obtain the highest yield of IAA by the strain I69, which was higher than that obtained by the combination of this bacterial isolate with another one (I22 + I69). Otherwise, a better result compared to previous studies concerning incubation time and L-Trp concentration (1 day of incubation and L-Trp concentration of 1 g/l) was detected. Moreover, the incubation temperature obtained by the used central composite design (36 °C) was close to that obtained by Leong [55]. The predictive response generated by the validated model under optimal parameters and the real obtained yield under these conditions were so close. We can, therefore, conclude that the precision of this approach has been well checked.

## Conclusion

Indole-3-acetic acid is one of the main physiological active hormones controlling various important processes in plants. In this study, bacterial isolates were screened in order to determine their ability for IAA production. Then, the inoculation effect by isolates in pure culture or in combination with *Acacia cyanophylla* seed’s germination was studied using the best performing isolates in terms of IAA production. The optimization of this phytohormone’s production was evaluated using a response surface methodology based on the central composite design on five factors. This method allowed us to determine the optimal conditions necessary to obtain the best IAA production (166 μg/ml). This yield, which exceeds that obtained by the synergy between the best strains producing IAA, has been experimentally verified. Finally, this study has shown that experimental designs provide a fast and relevant approach that can be used for optimizing the production of other phytohormones obtained from different strains.

## Data Availability

All data generated or analyzed during this study are included in this published article

## Abbreviations

FeCl_3_: Iron trichloride
H_2_SO_4_: Sulfuric acid
HClO_4_: Perchloric acid
HgCl2: Mercury Chloride
IAA: Indole-3-Acetic Acid
MgSO_4_, 7H_2_O: Magnesium sulfate heptahydrate
NaCl: Sodium chloride
PGPR: Plant Growth Promoting Rhizobacteria
pH: Potential hydrogen
RPM: Rotation per minute
Trp: Tryptophan
YMA: Yeast-Mannitol-Agar
α: Alpha
